# Effect of fixed-dose intravenous dexmedetomidine on emergence delirium after general anesthesia for a surgery in pediatric patients – A randomized controlled trial

**DOI:** 10.5339/qmj.2025.95

**Published:** 2025-08-20

**Authors:** Anwar ul Huda, Mohammad Yasir, Mohammad Zulqarnain Mughal, Asim Arif

**Affiliations:** 1 Security Forces Hospital, Riyadh, Kingdom of Saudi Arabia *Email: hudaanwar90@yahoo.com

**Keywords:** Dexmedetomidine, emergence delirium, anxiety, pediatric patients

## Abstract

**Background:**

Emergence delirium (ED) in pediatric patients can result in bodily harms, maladaptive changes, and longer post-anesthesia care unit (PACU) stays. The incidence of ED varies in pediatric patients depending on various factors such as age, type of anesthesia, type of surgery, pain, and the choice of diagnostic tools. Various pharmacological and non-pharmacological methods have been used to reduce its incidence postoperatively. This study aims to investigate the role of a fixed dose of intravenous dexmedetomidine in preventing ED in pediatric patients.

**Methods:**

Approval from the institutional ethical committee was obtained for this randomized controlled trial. Inclusion criteria included pediatric patients aged between 2 and 12 years, with ASA scores ranging from 1 to 3, who were scheduled to undergo general anesthesia for a surgical procedure. The intervention group received 20 ml of 0.2 mcg/kg dexmedetomidine intravenously over a period of 20 minutes before the end of the operation. In contrast, the control group received 20 ml of 0.9% saline. The primary outcome measure of this study was the incidence of ED in the PACU. All data collected during the study were entered and analyzed using the SPSS 22.0 statistical package program.

**Results:**

A total of 66 patients were included in the study. All baseline characteristics of both groups were similar. The incidence of ED in the control group was 42% (14/33), whereas it was 15% (5/33) in the dexmedetomidine group (*p* = 0.014).

**Conclusion:**

The use of 0.2 mcg/kg intravenous dexmedetomidine reduces the incidence of ED in patients undergoing general anesthesia with sevoflurane.

## 1. INTRODUCTION

Emergence delirium (ED) is an altered state of consciousness that usually occurs within 45 minutes after the cessation of anesthesia. It typically presents as disorientation, averted eyes or staring, psychomotor agitation and non-purposeful, resistive movements such as pulling, kicking, or flailing.^1,2^ ED can result in potential risks of bodily harm to both patients and healthcare staff, prolonged post-anesthesia care unit (PACU) stay, and postoperative maladaptive changes including temper tantrums, attention-seeking behavior, sleep alterations, and bedwetting in children.^2^

Risk factors for ED include preoperative anxiety and confusion, psychological immaturity, and the administration of various medications perioperatively.^2,3^ The incidence of ED is influenced by various factors such as the patient’s age, preoperative anxiety levels, the anesthesia technique used, the type of surgical procedures performed, the level of pain experienced, and the tools selected to diagnose ED. It is two to three times more prevalent in children compared to adults. Research indicates that the incidence of ED in pediatric anesthesia ranges between 20 and 80%.^4,5^

Various pharmacological interventions have been documented in the literature for the prevention of ED during the perioperative period, which include the administration of anesthetics such as propofol, fentanyl, ketamine, clonidine, midazolam, and dexmedetomidine.^5^ Dexmedetomidine is a potent, highly selective alpha-2 agonist. Its effect on brain receptors induces sedation similar to non-REM sleep with minimal respiratory depression.^6^ It has been used either as a continuous intravenous (IV) infusion or in fixed doses ranging from 0.15 to 2 mcg/kg to prevent ED in children.^7–9^ Although higher doses are associated with improved prevention of ED, they also lead to increased hemodynamic disturbances and longer PACU stays,^9^ whereas lower doses have proven to be less effective.^7^ The aim of this study was to investigate the role of a fixed dose of 0.2 mcg/kg dexmedetomidine in preventing ED in pediatric patients undergoing general anesthesia for elective surgeries.

## 2. METHODS

Approval from the institutional ethical committee was obtained (trial registration ID: NCT05813106 (Clinicaltrials.gov)). We included children aged between 2 and 12 years who were scheduled to undergo general anesthesia for surgery with an ASA score ranging from 1 to 3. Written informed consent for participation in the study was obtained from the parents or guardians of the patients. We excluded children whose parents refused enrollment or later requested their removal from the study, those unable to provide informed consent, and patients with known allergies to dexmedetomidine, psychiatric disorders, or those taking psychiatric medications.

Upon admission to the ward, a random ID was assigned to each patient who met the inclusion criteria. A computer-generated random number table was used for this purpose. Sealed opaque envelopes were used to determine the study groups once the patient arrived in the operation theater. The envelope was then handed to the anesthetist who was solely responsible for preparing the study medication. The primary anesthetist in the room was oblivious of the group allocation. Patients were assigned to one of two groups: group A – saline group (placebo) and group B – dexmedetomidine group. Perioperative anesthesia was standardized in both groups. The random ID assigned to each patient was used for collecting all patient data in the ward postoperatively.

The anxiety levels of each child were evaluated using the mYPAS (Modified Yale Preoperative Anxiety Scale) in the presence of one parent at 30 minutes before the commencement of surgery. This scale was initially developed in 1995^10^ and later modified in 1997.^11^ The mYPAS contains five items: activity, vocalizations, emotional expressivity, state of apparent arousal, and use of parent. Each item has Likert-type response options that reflect various behaviors. Children’s behavior is rated on a scale from 1 to 4 or 1 to 6, depending on the item. The score is then calculated by dividing each item rating by the highest possible rating for that item, followed by summing all the resulting values. This total is then divided by 5 and multiplied by 100. The resulting score ranges from 22.92 to 100, with higher scores implying greater anxiety. A cut-off score of 30 is used to determine the presence of high level of anxiety. The mYPAS demonstrates strong internal reliability, interrater reliability, and convergent validity.^11^

The technique of general anesthesia was identical in both study groups. Standard monitoring, including pulse oximetry, electrocardiography, and noninvasive arterial pressure, was performed for the patient on arrival to the operation room. Baseline heart rate, systolic and diastolic blood pressures, and mean arterial pressure were recorded before the initiation of anesthesia. Induction was performed using propofol at a dosage of 1–2 mg/kg, fentanyl at 1–2 mcg/kg, and rocuronium at 0.6 mg/kg. Sevoflurane was administered at 0.8–1.0 MAC (minimum alveolar concentration) to maintain anesthesia. Depending on the type of surgery, either a standardized caudal block or a remifentanil infusion at a dose of 0.05–0.2 mcg/kg/minute was used for intraoperative analgesia. All patients received 0.15 mg/kg dexamethasone and 15 mg/kg paracetamol intravenously.

The study drug was administered approximately 30 minutes before the end of the operation. In group A, patients received IV 20 ml of 0.9% saline (labeled as the study drug), while in group B, patients received 0.2 mcg/kg dexmedetomidine diluted with 20 ml of 0.9% saline (labeled as the study drug) over 20 minutes. At the end of the operation, any residual neuromuscular blockade was reversed using IV sugammadex at the dosage depending on the train of four readings. The tracheal tube was extubated after achieving adequate spontaneous regular breathing, gag reflex, facial grimaces, or eye opening and purposeful movements. All patients were then transferred to the PACU for routine monitoring. Emergence agitation was scored by using a standardized scoring system known as the Pediatric Anesthesia Emergence Delirium (PAED) score, which was recorded at the time of extubation and every 15 minutes until the patients were discharged from the PACU to the ward. The PAED score is considered a reliable and accurate tool for diagnosing ED in pediatric patients. This scale consists of five items that reflect consciousness, cognition, emotion, and psychomotor behaviors of pediatric patients.^12^

Pain scores were measured in PACU using a numerical rating scale (NRS). Patients with an NRS score ≥5 were managed using IV pethidine at doses of 0.2–0.3 mg/kg in boluses up to 1 mg/kg. Total opioid consumption, measured in milligrams, was documented in the PACU. Patients were also monitored for any other adverse events, such as bradycardia, hypotension, tachycardia, and hypertension, both intraoperatively and postoperatively. Postoperative nausea/vomiting (PONV) was graded on a scale from 0 to 3 based on severity. Patients were discharged from the PACU after they met the criteria established by the modified Aldrete score.

We used the 60% incidence of ED reported by Nair et al.^13^ for our sample size calculation. The ClinCalc.com sample size calculator was used. The sample size was found to be 60, with an anticipated 35% reduction in the incidence of emergence agitation due to the administration of IV dexmedetomidine, using an 80% power and a significance level of 0.05. Considering a 10% dropout, we determined our final sample size to be 66, with 33 participants allocated to each group.

All study data were entered into the statistical package for social sciences (SPSS) version 25 (SPSS Inc., Chicago, IL, USA). Continuous data are presented as mean ± standard deviation, while categorical data are presented as numbers or frequencies. Chi-square and Fisher’s exact tests were performed for categorical variables, and the Mann–Whitney U test was performed for continuous variables. A p value of less than 0.05 was considered as statistically significant.

## 3. RESULTS

**Figure 1:**
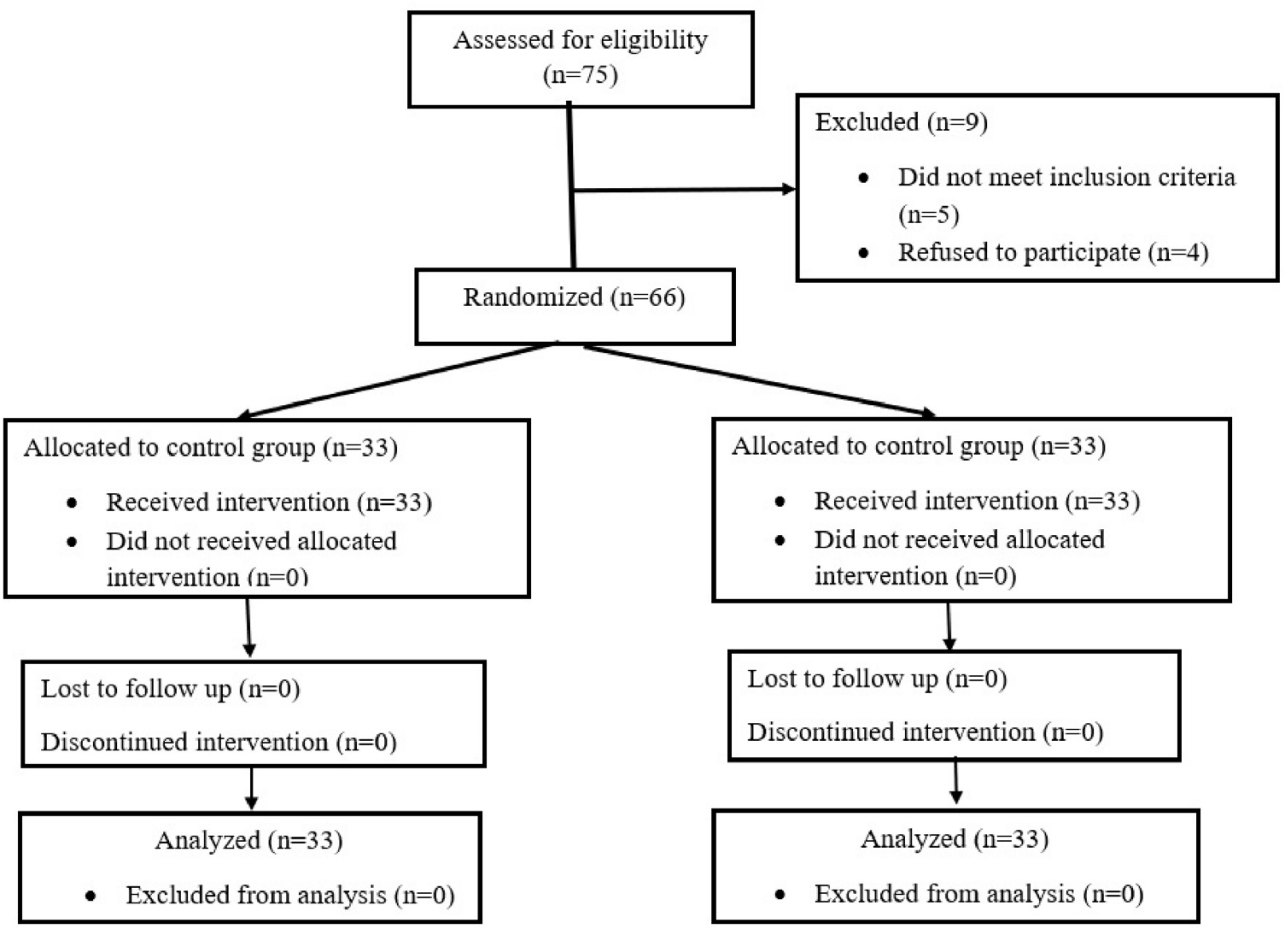
CONSORT flow diagram.

In this study, participants were recruited and followed up between December 1, 2022 and March 30, 2023. A total of 75 patients were assessed for eligibility; however, nine were excluded from the study. Specifically, five patients did not meet the inclusion criteria and four declined to participate. Ultimately, 66 patients were included in the study and randomly assigned to two groups, with 33 participants in each: group A – saline group (placebo) and group B – dexmedetomidine group. The CONSORT diagram is shown in Figure 1.

All variables were not found to exhibit a normal distribution. The baseline characteristics of the two groups were similar, as shown in Table 1.

**Figure 2:**
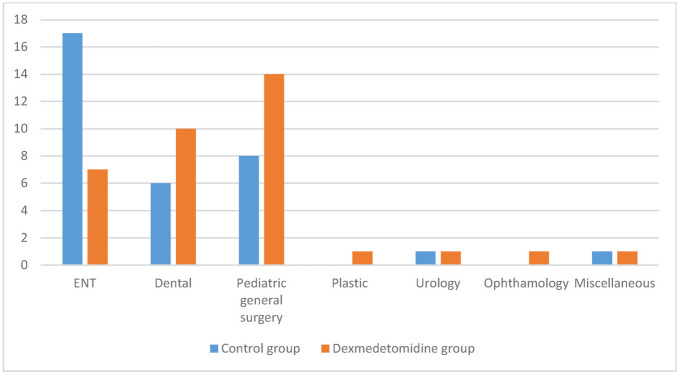
Distribution of cases in different surgical specialties.

The distribution of cases in both groups, according to surgical specialties, is shown in Figure 2.

The incidence of ED in the control group was 42% (14/33), in contrast to 15% (5/33) in the dexmedetomidine group (*p* = 0.014). Only two patients in the control group and one patient in the dexmedetomidine group received IV pethidine for pain relief.

## 4. DISCUSSION

Our study demonstrated that the incidence of ED in pediatric patients was 42% in the control group, while the administration of IV 0.2 mcg/kg dexmedetomidine 30 minutes before the end of the operation resulted in a significant decrease in ED to only 15%. In a separate study conducted by Jooma et al.,^14^ the incidence of ED was reported to be 51.6% in children undergoing dental surgery.^14^ Pieters et al.^15^ also reported an incidence of ED at 53%, which is higher than that observed in our control group. Various factors may account for the discrepancies in incidence rates across different studies. These studies have used different measuring tools and criteria for diagnosing ED. Commonly used scales include the Cravero scale, Watcha scale, PAED score, etc. The Watcha scale is considered a user-friendly tool for detecting delirium, whereas the PAED score is used to determine the severity of ED.^15–18^ The PAED score has previously been regarded as the gold standard for assessing ED; however, it has been modified by Locatelli et al.^19^ to increase its specificity by focusing only on the first three criteria.

There is no definite pathophysiology for the development of ED; however, it may arise due to alterations in the brain’s homeostatic processes.^20^ Additionally, the longer duration of surgery and anesthesia, along with rapid emergence from anesthesia, appear to be important factors contributing to ED.^21,22^ Several non-pharmacological interventions have been previously used to reduce the incidence of ED. There is a strong association between the occurrence of ED and the preoperative anxiety experienced by children, indicating that techniques aimed at reducing anxiety can be extremely beneficial.^2^ Such techniques include familiarizing with the operation room preoperatively, using video during mask induction to divert the child’s attention, using tablet-based interactive distractions, or playing a recorded voice of the mother during the arousal from general anesthesia.^23,24^

Pharmacological agents aimed at preventing ED in children, including opioids, total intravenous anesthesia (TIVA), ketamine, benzodiazepines, and dexamethasone, exhibit varying degrees of success.^5^ Among the opioids, fentanyl, sufentanil, remifentanil, and alfentanil have been used to prevent ED. However, the role of opioids in this context remains inconclusive. Intravenous fentanyl administered at a dose of 1–2 mcg/kg has been shown to decrease both the duration and severity of ED without extending the length of stay in the PACU or the hospital. However, it significantly increases the PONV. Additionally, it has been shown that opioids may reduce pain manifestations, potentially leading to an inaccurate estimation of the PAED score in children.^25^

Intravenous ketamine, given as a 1 mg/kg bolus followed by an infusion of 1 mg/kg/hour, has been found to be an effective preventive strategy against ED. However, it is associated with adverse effects such as nausea, vomiting, confusion, and hallucinations. High doses of ketamine have been associated with a 5–30% incidence of nightmares and hallucinations.^26^

The use of midazolam as a premedication or administered at the end of the operation has been extensively studied for preventing ED. However, its beneficial role has been questioned in a meta-analysis conducted by Mason.^27^ Recently, the use of TIVA has gained popularity across various surgical procedures due to its vested benefits, and Kanaya et al.^28^ demonstrated its positive role in preventing ED. Aouad et al.^29^ showed that even a single dose of 1 mg/kg propofol at the end of sevoflurane anesthesia can significantly decrease the occurrence of ED. However, in a comparative study, Ali and Abdellatif^30^ demonstrated that the pre-extubation use of IV dexmedetomidine at a dose of 0.3 mcg/kg was more effective than a single dose of propofol in preventing ED after adenotonsillectomy. The dose of dexmedetomidine used in their study was higher than our dose of 0.2 mcg/kg. Yao et al.^31^ reported a positive role of preoperative intranasal dexmedetomidine at a dose of 1–2 mcg/kg in preventing ED. In another study, Shukry et al.^32^ used a continuous infusion of 0.2 mcg/kg/hour intraoperatively in children undergoing general anesthesia with sevoflurane. Their findings showed a significant decrease in the incidence of ED in the dexmedetomidine group (*p* = 0.03). The number of ED episodes was also lower in the dexmedetomidine group (*p* = 0.017). Furthermore, there were no significant differences in pain scores, extubation time, and PACU stay between the two groups. Ghai et al.^7^ used two different dosages of IV dexmedetomidine, specifically 0.15 mcg/kg and 0.3 mcg/kg, in children undergoing cataract surgery. They demonstrated that a dose of 0.3 mcg/kg, which was higher than the dose used in our study, effectively reduced the incidence of ED from 35% in the saline group to zero in the dexmedetomidine group. Furthermore, this dosage did not increase side effects such as oversedation, bradycardia, hypotension, or prolonged PACU stays. However, the lower dose (0.15 mcg/kg) used in their study reduced the incidence of ED to 10% without delaying discharge time. Their results were inconsistent with those reported in the study conducted by Bong and Ng,^8^ who found insignificant difference in the incidence of ED after general anesthesia for MRI when using 0.3 mcg/kg dexmedetomidine intravenously.

Our study did not find any significant differences in the occurrence of adverse events such as hemodynamic events or PACU delays. Conversely, a meta-analysis conducted by Chen et al.^33^ demonstrated a significant decrease in ED, but at the expense of extended recovery times. This phenomenon may be attributed to relatively higher dosages used in most of the randomized controlled trials included in the meta-analyses. In another study conducted by Patel et al.,^9^ the authors used a bolus of 2 mcg/kg IV dexmedetomidine followed by an infusion at a rate of 0.7 mcg/kg/hour in children undergoing adenotonsillectomy. The overall dosage of dexmedetomidine used in this regimen was much higher than the 0.2 mcg/kg dose used in our study. Their findings indicated that this dexmedetomidine regimen was more effective in reducing ED (18%) compared to a single dose of 1 mcg/kg IV fentanyl (45.9%). However, they found a significant decrease in heart rate and blood pressure in the dexmedetomidine group than in the fentanyl group (*p* < 0.001). Based on our results, and in comparison with other studies, a single dose of 0.2–0.3 mcg/kg seems to be the optimal dose for reducing the incidence of ED without increasing side effects.

Our study has several limitations. Firstly, it is a single-center study. We did not compare different dosages of dexmedetomidine in our study. Another limitation may be the difficulty in distinguishing between pain-related agitation behavior and ED in relatively younger children, although the pain scores in both groups were not significantly different in our study. We also did not follow up patients after their discharge from the PACU.

## 5. CONCLUSION

The use of 0.2 mcg/kg IV dexmedetomidine reduces the incidence of ED in children undergoing general anesthesia using sevoflurane. Furthermore, it does not increase the occurrence of adverse events intraoperatively and postoperatively.

## COMPETING INTERESTS

The authors have no conflicts of interest to declare.

## AUTHORS’ CONTRIBUTIONS

AH: Contributed to the concept, literature search, conduct of the study and data analysis, manuscript writing and editing, and provided final approval. MY, MM: Assisted with literature search, conduct of the study, manuscript editing, and gave final approval. AA: Involved in the conduct of the study, manuscript editing, and provided final approval.

## Figures and Tables

**Table 1.** Baseline characteristics of the two groups.

**Variables**	**Control (group A)**	**Dexmedetomidine (group B)**	***p***
Age (in months), median (IQR)	63 (41.25)	59 (31.75)	0.903
Male/female (%)	70/30	64/36	0.547
Day cases (%)	67	45	0.068
Preoperative anxiety (%)	45	39	0.40
